# Biomethanation of Harmful Macroalgal Biomass in Leach-Bed Reactor Coupled to Anaerobic Filter: Effect of Water Regime and Filter Media

**DOI:** 10.3390/ijerph15050866

**Published:** 2018-04-26

**Authors:** Heejung Jung, Jaai Kim, Changsoo Lee

**Affiliations:** School of Urban and Environmental Engineering, Ulsan National Institute of Science and Technology (UNIST), 50 UNIST-gil, Eonyang-eup, Ulju-gun, Ulsan 44919, Korea; hjjung1018@unist.ac.kr (H.J.); jaai@unist.ac.kr (J.K.)

**Keywords:** anaerobic digestion, anaerobic filter, leach-bed reactor, microbial community structure, *Ulva*, water replacement

## Abstract

*Ulva* is a marine macroalgal genus which causes serious green tides in coastal areas worldwide. This study investigated anaerobic digestion as a way to manage *Ulva* waste in a leach-bed reactor coupled to an anaerobic filter (LBR-AF). Two LBR-AF systems with different filter media, blast furnace slag grains for R1, and polyvinyl chloride rings for R2, were run at increasing water replacement rates (WRRs). Both achieved efficient volatile solids reduction (68.4–87.1%) and methane yield (148–309 mL/g VS fed) at all WRRs, with the optimal WRR for maximum methane production being 100 mL/d. R1 maintained more stable methanation performance than R2, possibly due to the different surface properties (i.e., biomass retention capacity) of the filter media. Such an effect was also noted in the different behaviors of the LBR and AF between R1 and R2. The molecular analysis results revealed that the development of the microbial community structure in the reactors was primarily determined by the fermentation type, i.e., dry (LBR) or wet (AF).

## 1. Introduction

Anaerobic digestion (AD) has been extensively used to treat organic waste because it can produce energy while removing pollutants. Recently, its role in diversifying energy sources is gaining increasing attention. Producing methane-rich biogas from waste biomass through AD is considered a viable option for sustainable energy supply. In this context, harmful and/or inedible macroalgae have emerged as a feasible feedstock for biogas production. Macroalgal biomass is rich in biodegradable organics and contains little lignin, which is beneficial for its efficient bioconversion. *Ulva* is a macroalgal genus noted for causing harmful green tides worldwide [1,2]. Green tides have significant environmental and economic consequences related to the accumulation and decay of bloomed algal biomass [3]. Given that macroalgal blooms have become more frequent and severe with global warming, biomethanation of *Ulva* biomass appears to be environmentally and economically attractive.

Hydrolysis often becomes the rate-limiting step of the overall AD process when treating feedstocks with high suspended solids content such as macroalgae. *Ulva* biomass has often been mechanically ground or milled before feeding to improve the rate of hydrolysis [4,5,6]. With its simple and flexible configuration, a leach-bed reactor (LBR) is accepted as a suitable design for dry or high-solids AD (>15–20% total solids) [7,8]. Compared to wet AD, dry AD using LBR can reduce reactor volume and energy consumption for heating and mixing [9]. An LBR can be coupled to an attached-growth reactor—e.g., an upflow anaerobic sludge blanket (UASB) reactor or anaerobic filter (AF)—for high retention of microorganisms and thus high AD performance [9,10]. In such two-stage systems, leachate from the first stage (i.e., LBR optimized for hydrolysis/acidogenesis) is fed to the second stage for methanogenesis, which enables efficient AD of high-solids feedstocks with minimal pretreatment. One of the major challenges in operating LBR is how to prevent channeling and enhance solubilization. Viable approaches to these issues include recirculating leachate or effluent and replacing leachate with water, which helps in solubilizing particulate organic substances by increasing water content, redistributing hydrolytic microorganisms and enzymes, and improving mass transfer [7,11].

A high microbial density is achieved in AFs by forming biofilms on the media surfaces and trapping suspended microorganisms within the filter bed, helping the reactor maintain a stable performance under high organic loading conditions [12,13]. The physicochemical properties of filter media—such as specific surface area, surface morphology, porosity, grain size, and bulk density—significantly affect the microbial retention capacity and thus the process performance of an AF [14]. Different media—such as gravel, shells, rubber sheets, and plastic rings—have been applied in AFs to treat different waste streams [13,15]. This study investigated two LBR-AF systems with different filter media for the AD of *Ulva* biomass: one filled with blast-furnace slag (BFS) grains and one with polyvinyl chloride (PVC) rings. Their performances were compared at different water replacement rates (WRRs) of the leachate to examine the potential of the LBR-AF system for treating *Ulva* biomass and the effects of different filter media on biomethanation efficiency. In contrast to most previous studies reporting the effect of WRR on organic leaching in single-stage LBRs [7,11,16], this study was conducted in two-stage LBR-AF systems. Additionally, the underlying microbial communities were analyzed by a combination of molecular and statistical approaches to better understand the systems’ behavior.

## 2. Materials and Methods

### 2.1. Inoculum and Ulva Biomass

The anaerobic sludge used as inoculum for the experimental systems was collected from a local biogas plant co-digesting food waste and sewage sludge. Naturally occurring *Ulva* biomass collected from a local beach was rinsed with tap water to remove impurities and stored in a freezer until use. Physicochemical characteristics of the *Ulva* substrate and the inoculum are presented in Table 1.

### 2.2. Reactor Setup and Operation

**Figure 1:**
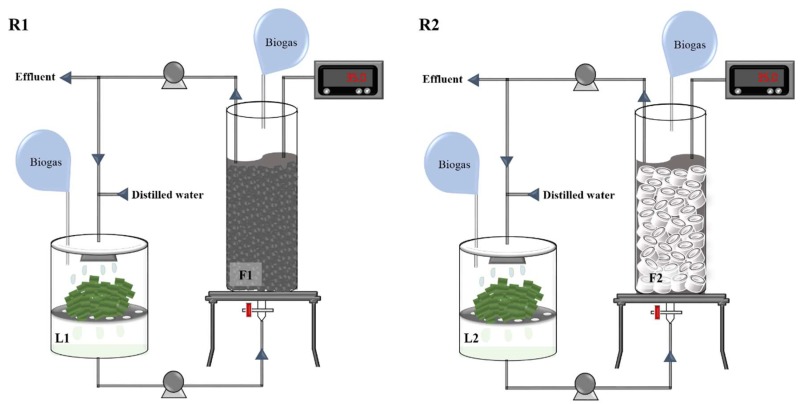
Schematic of the experimental LBR-AF systems with BFS grains (R1) or PVC rings (R2) as filter media. Leachate from each LBR was pumped into the bottom of the coupled AF, and the effluent from the AF was recirculated to the LBR.

Two LBR-AF systems—namely, R1 and R2—were configured as illustrated in Figure 1. The LBRs (i.e., L1 for R1 and L2 for R2) consisted of a 1.4-L cylindrical plastic vessel (10.5-cm wide and 15-cm high). The thawed *Ulva* biomass was chopped into approximately 3–4 cm pieces without further mechanical disintegration by grinding or milling and loaded into a wire mesh basket 6 cm above the bottom of each LBR. *Ulva* leachate percolated through the mesh was collected at the bottom. Each LBR was initially loaded with 250 g of substrate, and 250 mL of distilled water (DW) [17] was added to the top. Leachate from each LBR was pumped into the bottom of the corresponding AF (i.e., F1 for R1 and F2 for R2), and the AF effluent was recirculated to the LBR. The AFs consisted of a glass column (9-cm wide and 60-cm high) packed with filter media (BFS grains for F1 and PVC rings for F2) to 85% of the column height. BFS grains of 5–10 mm in size were prepared as previously described [18], while PVC rings were prepared by cutting 6-mm thick PVC pipes (internal diameter, 1.3 cm) to 2 cm in length. The specific surface area and bulk density of the BFS grains were 2.37 m^2^/g and 2.30 g/cm^3^, respectively, while those of the PVC rings were 0.16 m^2^/g and 1.49 g/cm^3^, respectively. More detailed information on the physicochemical characteristics of the BFS grains are available in a previous study [18]. Both filter media were washed repeatedly with tap water to remove impurities before use. A perforated steel plate was placed at the bottom of AFs to support the filter bed and to allow for even distribution of the influent flow. The AFs were initially filled with anaerobic sludge to the height of the filter bed (void volume, 1.1 L for F1 and 1.6 L for F2) and starved with internal circulation for a week until biogas production ceased, before its coupling to an LBR.

The LBR-AF systems were tested in batch mode at four WRRs of 50, 100, 150, and 200 mL/d. Water replacement was performed by drawing off a volume of the AF effluent and adding the same volume of DW into the LBR once a day. The AFs were operated at 35 ± 2 °C, while the LBRs were run at room temperature without temperature control. The effluent recirculation from AF to LBR occurred at a continuous flow rate of 10 mL/min by peristaltic pumps. The LBR leachate and AF effluent samples were periodically analyzed to monitor system performance. At each WRR, the LBR-AF systems were run until biogas production ceased. The *Ulva* biomass loss in total solids (TS) and volatile solids (VS) was measured for each batch cycle by subtracting the amount of *Ulva* residue left in the LBR from that of *Ulva* biomass initially loaded into the reactor. This was defined as the substrate removal efficiency. Biogas production from each reactor was measured by water displacement and corrected to standard temperature and pressure (0 °C and 1 bar).

### 2.3. DNA Extraction

Biomass samples for microbial community analysis were collected from the *Ulva* residue (in L1 and L2) and the AF biofilm (in F1 and F2) at the end of each WRR cycle. A portion of the *Ulva* residue left in the LBR was weighed and diluted 10-fold (w/v) with DW, and then the microorganisms attached to the substrate surface were released by vigorous vortexing for 5 min. A 1-mL aliquot of mixed liquor was taken from each AF at the midpoint of the filter bed through a sampling port. A 1-mL aliquot of each biomass sample was washed by repeated centrifugation and resuspension as previously described [19]. A 200-L aliquot of the final suspension was loaded on an ExiProgen nucleic acid extractor (Bioneer, Daejeon, Korea) for the extraction of total DNA following the manufacturer’s instructions. The recovered DNA was eluted in 100 L of elution buffer and stored at −20 °C until use.

### 2.4. Real-Time Polymerase Chain Reaction

Real-time polymerase chain reaction (PCR) was conducted to determine the abundance of bacteria, methanogens, and sulfate-reducing bacteria (SRBs). The absolute concentrations of total bacteria and five methanogen groups (i.e., *Methanobacteriales*, *Methanomicrobiales*, *Methanococcales*, *Methanosarcinaceae*, and *Methanosaetaceae*) were analyzed by targeting the 16S rRNA gene as previously described [20]. The concentration of SRBs was measured by real-time PCR with a primer set targeting the dissimilatory sulfite reductase A gene (*dsrA*) ubiquitous in all SRBs [21]. The real-time PCR reactions for *dsrA* quantification were prepared using the THUNDERBIRD SYBR qPCR Mix (TOYOBO, Osaka, Japan) and amplified in a QuantStudio 12K Flex system (Life Technologies, Carlsbad, CA, USA) as previously described [22].

A standard curve was constructed for each target microbial group as previously described using an equimolar mixture of the corresponding standard plasmids [23]. Three standard plasmids carrying one copy of *dsrA* were generated by cloning the PCR amplicons from reactor samples with different melting profiles (i.e., different sequences) into pGEM-T Easy vectors (Promega, Madison, WI, USA). The copy concentration of a target sequence in unknown samples was quantified against the corresponding standard curve. Each sample was analyzed in duplicate.

### 2.5. High-Resolution Melting Analysis

High-resolution melting (HRM) analysis was performed using a QuantStudio 12K Flex system (Life Technologies). Archaeal and bacterial 16S rRNA genes were amplified using the same primers and thermal protocol as for the real-time PCR. An HRM reaction mixture (20 L) was prepared using the MeltDoctor HRM Master Mix (Life Technologies) as previously described [19]. The resulting amplicons were denatured (95 °C, 10 s) and renatured (60 °C, 1 min), and then their melting properties were analyzed while increasing the temperature from 60 °C to 95 °C at a rate of 0.015 °C/s. Melting peak plots were generated based on the melting profiles, within a melting region of 80.0–88.4 °C for archaea and of 78.1–88.3 °C for bacteria, using QuantStudio 12K Flex software ver. 1.2 (Life Technologies) as previously described [19]. Each sample was analyzed in duplicate.

The resulting archaeal and bacterial melting peak profiles were each transformed to a matrix as previously described with minor modifications [19]. The dissociation rates (−dR_n_/dT) were normalized to their sum within the melting region for HRM analysis. A relative abundance matrix was constructed by taking the relative dissociation rates at intervals of 0.2 °C (i.e., separation resolution). To analyze the relatedness between the reactor microbial community structures, cluster analysis with the unweighted pair group method with arithmetic means (UPGMA) algorithm and nonmetric multidimensional scaling (NMS) were conducted on the obtained matrices using Sorensen distance measure [24]. Computations for clustering analysis and NMS ordination were performed using PAST 3.14 (http://folk.uio.no/ohammer/past/) and PC-ORD 6 software (MjM Software, Gleneden Beach, OR, USA), respectively.

### 2.6. Analytical Methods

Chemical oxygen demand (COD) was measured colorimetrically using an HS-COD-MR kit (HUMAS). Volatile fatty acids (VFAs) were determined using a gas chromatograph (7820A, Agilent, Santa Clara, CA, USA) equipped with a flame ionization detector and an Innowax column (Agilent). Samples for soluble COD and VFA measurements were prepared by filtration through a 0.45 m pore membrane filter. Biogas composition (CH_4_, CO_2_, and H_2_) was analyzed by another 7820A gas chromatograph coupled with a thermal conductivity detector and a ShinCarbon ST column (Restek, Bellefonte, PA, USA). The H_2_S content in the biogas was measured using a 7890A gas chromatograph (Agilent) equipped with a flame photometric detector and an HP-1 column (Agilent). The C, H, O, N, and S contents of *Ulva* biomass (dry weight basis) were analyzed using a Flash 2000 elemental analyzer (Thermo Scientific, Waltham, MA, USA). Solids were determined according to the Standard Methods [25]. All analyses were performed at least in duplicate.

## 3. Results

### 3.1. Degradation of Ulva Biomass

**Figure 2:**
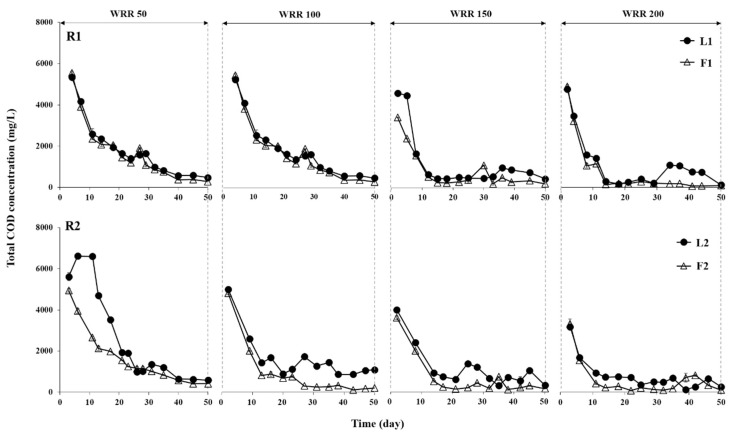
Total COD profiles of the LBR and AF in R1 and R2 during the batch experiment at each WRR (mL/d).

**Figure 3:**
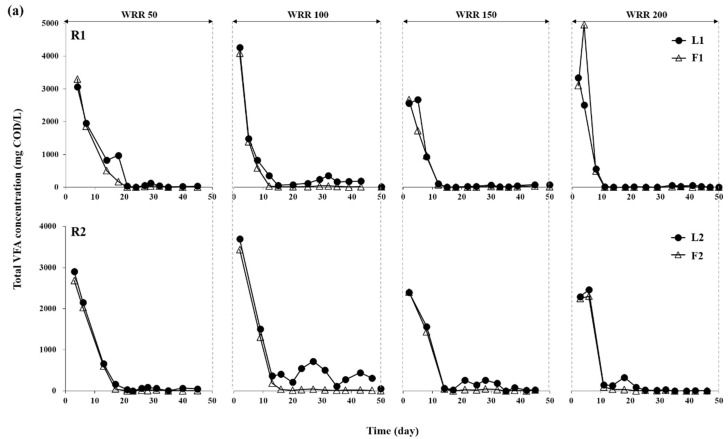

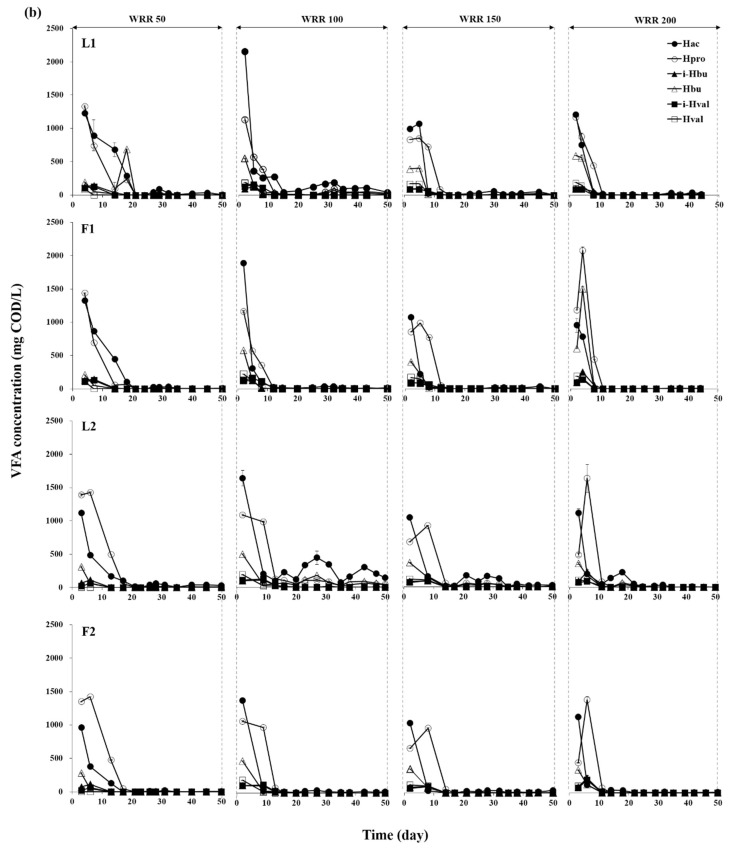
Total (**a**) and individual (**b**) VFA profiles of the LBR and AF in R1 and R2 during the batch experiment at each WRR (mL/d).

The effect of water regime on the performance of the LBR-AF systems was investigated at four different WRRs. *Ulva* biomass was effectively degraded in both R1 and R2 during the batch experiment at all WRRs (Figure 2). The high leachate and effluent COD concentrations in the initial phase of operation at all WRRs in both systems reflect the fast hydrolysis of readily biodegradable organics in the substrate. Both of the COD levels decreased rapidly with time due to the removal of organic matter by water replacement and methanogenic conversion. In both systems, VFAs—particularly acetate, propionate, and butyrate—formed the majority of the soluble COD in the leachate and effluent at all WRRs (Figure 3). The leachate and effluent pH remained above 6.3 and 6.8, respectively, throughout the experiment in R1 and R2. Similar to the COD profiles, VFAs accumulated initially with the fast acidogenesis of *Ulva* biomass and decreased rapidly to the bottom at all WRRs. Interestingly, the LBR and AF reactors paired in a system (i.e., L1 and F1 for R1 and L2 and F2 for R2) showed very similar COD and VFA profiles, indicating that they behaved as a single-stage reactor in terms of bulk liquid characteristics at the high rate of effluent recirculation between them (9–13 turnovers/d).

The highest effluent concentration of total VFAs was observed at a WRR of 100 mL/d in both R1 and R2, suggesting that this WRR may be optimal for efficient acidogenesis of *Ulva* biomass. As the composition of acidogenic intermediates significantly affects methanogenic performance, characteristics of the leachate are important factors for LBR operation [11]. The observation of acetate, propionate, and butyrate as the main acidogenic products (Figure 3b) agrees with previous LBR studies [8,11]. Acetate and butyrate are favorable intermediates for methanogenesis, and therefore, their dominance was likely beneficial for the efficient and stable biomethanation of *Ulva* biomass. Propionate is also a common acidogenic product in AD, but its accumulation is inhibitory to methanogenesis because its anaerobic degradation is thermodynamically unfavorable [26]. Although a temporary accumulation of propionate was observed in the early period at all WRRs, it degraded rapidly without apparent inhibition in both experimental systems.

The degree of substrate degradation at each WRR was determined by measuring the VS and TS reductions of the *Ulva* biomass fed to the LBR (Table 2). The observed TS and VS reductions were markedly higher than those from previous studies using LBRs without water replacement [7,9,11,27,28]. This indicates the beneficial effect of water replacement on the anaerobic degradation of *Ulva* biomass in LBR settings.

### 3.2. Methane Production Performance

**Figure 4:**
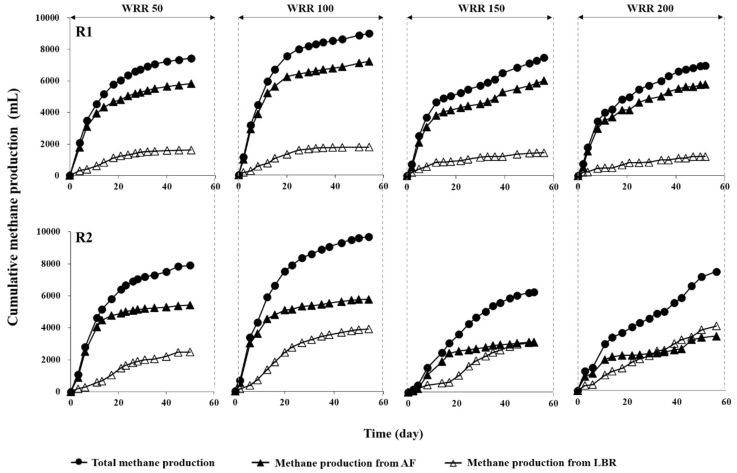
Cumulative methane production in R1 and R2 during batch experiment at each WRR (mL/d).

Both R1 and R2 showed effective methane production from *Ulva* biomass at all WRRs, indicating that the LBR-AF systems developed in this study were functioning properly. The water regime showed a significant effect on methane production, in terms of rate as well as yield, in both systems (Figure 4). The cumulative methane production increased in both systems with increasing WRR from 50 mL/d to 100 mL/d but declined with further increases above 100 mL/d. The variations in methane productivity with respect to the changes in WRR were more pronounced in R2 than in R1 (Table 2). The methane productions in R1 and R2 reached the maximum values of 8088 and 9613 mL, respectively, at a WRR of 100 mL/d. In contrast, the maximum methane production per total working volume (i.e., sum of the working volumes of LBR and AF) was 25.6% higher in R1 than in R2.

To take into account the loss of substrate organics by water replacement into account in evaluating the methane yield, the ‘true yield’ (Ymt) was estimated based on the amount of substrate organics retained and consumed within the system boundary, in addition to the ‘apparent yield’ (Yma), which was calculated without considering the organic loss. The Yma per unit mass of VS fed (Yma_f_) was 203–285 and 148–309 mL/g VS fed in R1 and R2, respectively, while the Ymt per unit mass of VS fed (Ymt_f_) was 225–317 and 161–344 mL/g VS fed in R1 and R2, respectively (Table 2). Both apparent and true methane yields were slightly higher in R2 than in R1 at WRRs of 50 and 100 mL/d. However, they were markedly higher in R1 than in R2 at further increased WRRs. The maximum yields were observed at a WRR of 100 mL/d in both systems and were 1.4- and 2.1-fold higher than the lowest yields in R1 and R2, respectively. The observed yields are higher than those from previous studies which used different two-stage systems consisting of an LBR coupled to a methanogenic reactor to treat various feedstocks, including energy crops and grass silage [7,9,27,28]. AD of *Ulva* biomass using LBR has rarely been studied, and little data is available for comparison. A recent study performed the co-digestion of brown and red seaweeds using a combination of an LBR and a UASB [29]. Although the biodegradability of green macroalgae, including *Ulva*, is reportedly lower than that of brown or red macroalgae [30,31], considerably higher methane yields (up to 1.9-fold based on the maximum Yma_f_) than those in the literature were observed in both R1 and R2.

Interestingly, a substantial amount of methane was produced not only in AF but also in LBR at all WRRs in both experimental systems (Figure 4). The contribution of LBR to methane production was significantly greater in R2 than in R1, and it exceeded that of AF at WRRs of 150 and 200 mL/d. The different behaviors can be attributed to the use of different filter media: BFS grains for F1 and PVC rings for F2. In R1, methane was primarily produced in F1 (78.3–82.7%) at all WRRs (Table 2). In R2, however, the methane production in F1 decreased with increasing WRR to account for 45.7% of the total production at a WRR of 200 mL/d. The methane production was significantly higher and varied more greatly in L2 (2482–4007 mL) than in L1 (1190–1736 mL), likely indicating that L2 maintained higher methanogenic activity than L1 at all WRRs.

### 3.3. Microbial Community Structures

**Figure 5:**
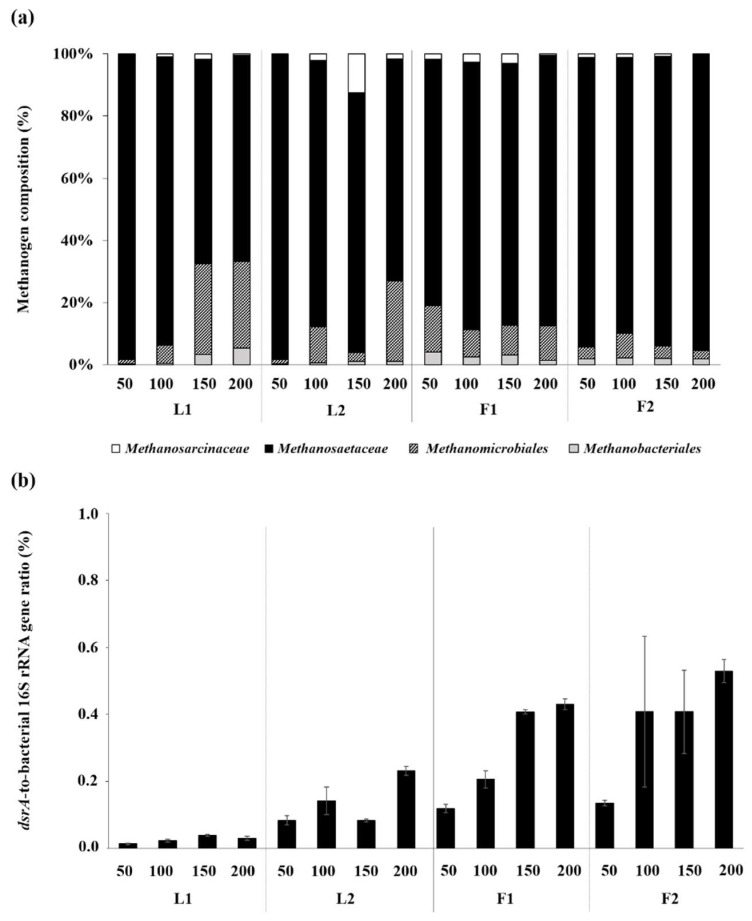
Methanogen community composition (**a**) and the *dsrA*-to-the-total bacterial 16S rRNA gene ratio (**b**) in the residual *Ulva* biomass in the LBR and the filter biomass in the AF. Samples are labeled with WRRs (mL/d) and the corresponding reactor names.

The real-time PCR results revealed that both LBR (residual *Ulva* biomass) and AF (filter biomass) were highly populated by bacteria and methanogens in both R1 and R2. In terms of 16S rRNA gene concentration, *Methanosaetaceae* was the predominant methanogen group in the filter biomass in both F1 (79–87% of the total methanogens measured (Met)) and F2 (89–95% of Met). Although at much lower levels, *Methanomicrobiales*, *Methanobacteriales*, and *Methanosarcinaceae* were also detected at all WRRs in both experimental systems. This means that methanogenesis occurred through both aceticlastic and hydrogenotrophic routes, with the former being the dominant pathway, in the AFs. The methanogen communities in the LBRs were also largely dominated by *Methanosaetaceae* at all WRRs, suggesting that the aceticlastic pathway was the primary route of methanogenesis in the LBRs. However, the methanogen composition changed much more radically in the LBRs than in the AFs (Figure 5a). The dominance of *Methanosaetaceae* decreased, while the fraction of hydrogenotrophic methanogens, particularly *Methanomicrobiales*, increased drastically with increasing WRR in both L1 and L2. This implies that increasing WRR led to the emergence of hydrogenotrophic methanogenesis while reducing the aceticlastic activity in the LBRs.

As members of the bacterial community, SRBs existed at a considerable level in all biomass samples analyzed (Figure 5b). This can be associated with the high sulfur content of *Ulva* biomass (Table 1). Although the proportion of SRBs to the total bacteria, determined by the ratio of *dsrA* to bacterial 16S rRNA gene concentrations, was as low as <1%, it was markedly higher in AF than in LBR at all WRRs in both R1 (up to 14.4-fold) and R2 (up to 4.9-fold). As a consequence of sulfate respiration by SRBs, the H_2_S content of the total biogas produced at a given WRR was as high as 0.2–0.6% (*v*/*v*) in the systems (data not shown).

**Figure 6:**
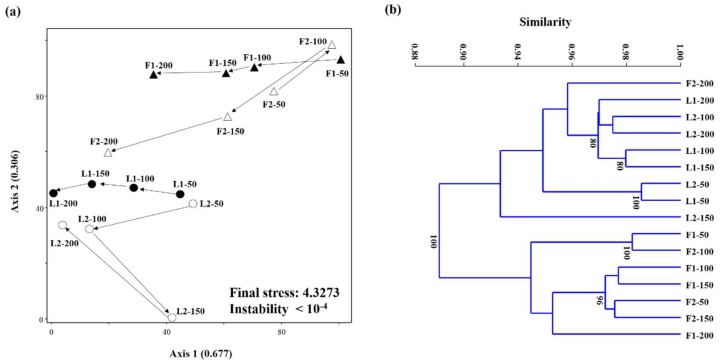
NMS plot (**a**) and cluster dendrogram (**b**) generated based on the archaeal HRM peak profiles from the residual *Ulva* biomass in the LBR and the filter biomass in the AF. Data points are labeled with the corresponding reactor names followed by WRRs. Bootstrap values higher than 70% (1000 replicates) are shown.

**Figure 7:**
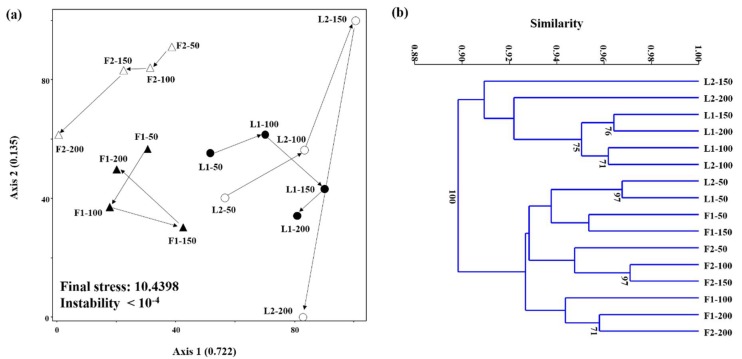
NMS plot (**a**) and cluster dendrogram (**b**) constructed based on the HRM peak profiles of bacterial 16S rRNA gene PCR products from residual *Ulva* biomass and filter biomass of R1 and R2. Data points are labeled with the corresponding sample types followed by WRRs (mL/d). Bootstrap values higher than 70% (1000 replicates) are shown.

The cluster dendrograms and NMS plots visualizing the changes in archaeal and bacterial community structures with increasing WRR based on the HRM data are shown in Figure 6 and Figure 7. The cumulative *r*^2^ for the ordination axes was 0.983 and 0.857 in the archaeal and bacterial NMS plots, respectively, suggesting that both plots explain most of the total variability in the community structure data sets. The final stress (<11) and instability (<10^−4^) were sufficient to guarantee reliable ordination results in both NMS plots [24]. Both archaeal and bacterial community structures largely clustered according to whether they were sampled from LBR (L1 and L2) or AF (F1 and F2). This indicates that both methanogen and bacterial community structures developed in different ways between the LBR and AF probably due to the different reactor designs and operating conditions. It is notable that R2 showed larger and more dynamic variations in microbial community structure with increasing WRR than R1.

## 4. Discussion

The overall results demonstrate that WRR had a significant influence on the performance of the LBR-AF systems in terms of leaching soluble organics and yielding methane from *Ulva* biomass. Although total VFAs accumulated to a considerable level (up to 5 g COD/L), the accumulation was temporary and completely stabilized during the operation at all WRRs in the experimental systems (Figure 3b). This implies the development of a robust syntrophic microbial consortium necessary for anaerobic oxidation of VFAs longer than acetate in both R1 and R2 [32]. Both systems showed the most favorable VFA composition (i.e., higher concentration of acetate) at a WRR of 100 mL/d. Correspondingly, the maximum methane production was observed at the same WRR in both systems (Figure 4). Further increase in WRR led to an increased fraction of propionate along with decreased methane production in both systems. These data suggest that 100 mL/d was the optimal WRR of both LBR-AF systems for the biomethanation of *Ulva* biomass. Rapid acidification that was inhibitory to the overall AD performance could occur during the initial period of operation in LBRs, and its adverse effect on the biomethanation of seaweeds has been previously reported [29,33]. Water replacement and effluent recirculation, as applied in this study, can provide a method to avoid such inhibition by facilitating the hydrolysis of substrate and diluting the leachate [8,34]. Meanwhile, an excessively high WRR can result in a considerable waste of substrate organics for methane production. Therefore, applying a proper WRR is important in an LBR for biomethanation. Interestingly, methane production occurred in the LBR as well as the AF at all WRRs in both R1 and R2 (Figure 4). This indicates that methanogenic activity developed in the LBRs at all WRRs due to the recirculation of AF effluents containing substantial amounts of methanogens and alkalinity [11,29].

Interestingly, in both experimental systems, the LBR as well as the AF were highly populated by bacteria and methanogens. This appears to support the observation of considerable methane production in the LBRs, whose primary function was solubilizing *Ulva* biomass, at all WRRs. The significantly greater contribution of the LBR to the overall methane production in R2 than in R1 may be explained by the different characteristics of the filter media between F1 and F2. It is generally understood that the physical properties of filter media (e.g., specific surface area, surface morphology, and porosity) have significant effects on cell attachment and biofilm growth, which is closely related to the methanogenic activity [12,14]. Although extensively used, PVC rings have a limitation as filter media related to their lower ability to retain microorganisms compared to high-specific-area material [14]. BFS is a by-product from iron ore processing and has recently been employed as AF media because of its porous and bulky structure [18,35]. The BFS grains used for F1 had a significantly higher specific surface area (2.37 m^2^/g) compared to the PVC rings used for F2 (0.16 m^2^/g). This suggests that F1 could be more effective in retaining biomass than F2, or, in other words, F2 is likely more vulnerable to biomass loss by hydraulic washout than F1. It seems therefore reasonable to infer that the filter biomass, inoculated with anaerobic sludge at the start of the experiment, is more easily removed from the filter bed and flew into the LBR in R2 compared to R1. This could explain why the functional separation of LBR and AF was blurred in R2, particularly at higher WRRs (i.e., higher hydraulic pressure), whereas their functions were relatively clearly separated (i.e., L1 for hydrolysis/acidogenesis and F1 for methane production) regardless of WRR in R1 (Figure 4). A portion of the biomass washed out of the AF is to be discarded through the effluent drawn off for water replacement, which may cause a significant decrease in methane productivity. The apparent decrease in methane production with increasing WRR above 100 mL/d in F2 can be attributed to such an effect. The higher biomass retention capacity of BFS may explain why F1 maintained greater methane productivity than F2, despite having a significantly smaller void volume (i.e., lower inoculum size). In contrast, the total methane production was largely comparable between R1 and R2 (<10% difference except at a WRR of 150 mL/d) due to the higher methane production in L2 than in L1 (Table 2).

Although the LBR-AF systems were operated with high-rate effluent recirculation (9–13 turnovers/d), both the LBR (dry fermentation) and AF (wet fermentation) showed apparently different microbial community structures (Figure 6 and Figure 7). It is further interesting that both archaeal and bacterial community structures were generally clustered according to whether they were from LBR or AF, rather than R1 or R2. This suggests that reactor type (i.e., fermentation type) was likely the dominant factor determining the development of microbial community structures. This may also be related to the different functions of the LBR and AF given that the functional properties of a biological process is directly linked to the underlying microbial ecology. It is notable that the archaeal community structure in F2 shifted gradually toward that in L2 with increasing WRR. This corresponds well to the increasing contribution of L2 to the overall methane production in R2, along with the decrease in methane production from F2 (Figure 4). Supportively, the archaeal community structure in F2 at the highest WRR was the only one from the AF biomass clustered together with those in the LBRs. Very little has been studied so far on the microbial ecology in LBRs, and this study provides a rare insight into it.

Another point to note is the production of considerable amounts of H_2_S (Table 2). This is an expectable consequence of using sulfur-rich *Ulva* biomass as substrate. The H_2_S content of the total biogas produced ranged 0.2–0.6% (*v*/*v*), which requires thorough purification [36]. It is therefore suggested that sulfide control is an important concern in the AD of *Ulva* biomass. The *dsrA*-to-bacterial 16S rRNA gene ratio was not directly proportional to the H_2_S production, although it somewhat reflected the higher sulfidogenic activity in AF and in LBR, particularly in R1. This may be due to the limitation of a DNA-based analysis to quantitatively reflect the metabolic activity, which may be more directly indicated by an RNA-based analysis. This may suggest further research into the quantitative indicators of sulfide productivity.

## 5. Conclusions

*Ulva* biomethanation was performed in two LBR-AF systems with different filter media, BFS grains and PVC rings at varying WRRs. Both systems achieved effective conversion of *Ulva* biomass to methane at all WRRs. The highest methane productivity was observed at 100 mL/d WRR in both systems. The different properties of the filter media, related to the ability to retain microorganisms, seem to have resulted in the different behaviors of the LBR and AF between R1 and R2. Fermentation type—i.e., dry (LBR) or wet (AF)—was likely the dominant factor affecting the microbial community structure development in the experimental systems.

## Figures and Tables

**Table 1:** Physicochemical characteristics of the inoculum and substrate used.

Parameters	Units	Anaerobic Sludge	*Ulva* Biomass
Total COD	g/L	27.5 (0.1) ^a^	- ^b^
Soluble COD	g/L	2.7 (0.0)	-
TS	g/L or g/kg wet ^c^	38.2 (0.1)	153.9 (8.4)
TVS	g/L or g/kg wet	22.6 (0.1)	126.1 (7.8)
TSS	g/L	30.8 (0.3)	-
VSS	g/L	20.5 (0.3)	-
Carbon	%	27.8 (0.5)	37.8 (0.5)
Hydrogen	%	4.4 (0.1)	7.2 (0.3)
Nitrogen	%	5.4 (0.1)	5.4 (0.0)
Sulfur	%	1.0 (0.2)	1.9 (0.2)
Oxygen	%	22.2 (0.6)	35.7 (0.3)

^a^ Standard deviations are in parentheses; ^b^ Not determined; ^c^ g/L for anaerobic sludge; g/kg wet for *Ulva* biomass.

**Table 2:** Performance of the experimental reactors at different water replacement rates.

Parameter ^a^	Unit	R1				R2			
		WRR 50 ^b^	WRR 100	WRR 150	WRR 200	WRR 50	WRR 100	WRR 150	WRR 200
TS reduction	%	85.0 (0.1) ^c^	75.4 (0.6)	75.9 (0.8)	69.3 (3.3)	78.4 (0.7)	76.3 (1.0)	73.1 (0.8)	73.3 (2.2)
VS reduction	%	87.1 (0.8)	78.0 (0.8)	75.4 (1.1)	68.4 (2.1)	82.3 (0.0)	78.4 (1.0)	72.9 (0.5)	71.7 (2.0)
Total CH_4_	mL	7437 (28)	8808 (16)	7415 (26)	6878 (14)	7905 (56)	9613 (43)	6165 (70)	7381 (35)
CH_4_ from LBR	mL	1614 (1)	1736 (8)	1438 (25)	1190 (6)	2482 (5)	3881 (10)	3066 (66)	4007 (1)
CH_4_ from AF	mL	5823 (28)	7072 (14)	5977 (8)	5688 (13)	5423 (55)	5732 (42)	3099 (22)	3374 (35)
Yma_f_	mL/g VS fed	203 (0.01)	285 (0.02)	210 (0.0)	238 (0.0)	216 (0.02)	309 (0.02)	148 (0.02)	218 (0.02)
Yma_t_	mL/g VS fed	225 (0.07)	317 (0.09)	231 (0.08)	280 (0.08)	236 (0.07)	344 (0.18)	161 (0.01)	251 (0.1)
Total H_2_S	mL	84.3 (0.0)	93.1 (0.0)	53.6 (0.0)	48.0 (0.0)	87.0 (0.0)	43.3 (0.0)	27.2 (0.0)	26.5 (0.0)
H_2_S from LBR	mL	16.7 (0.0)	10.8 (0.0)	10.0 (0.0)	8.9 (0.0)	23.2 (0.0)	21.8 (0.0)	13.3 (0.0)	13.7 (0.0)
H_2_S from AF	mL	67.6 (0.0)	82.3 (0.0)	43.6 (0.0)	39.1 (0.0)	63.8 (0.0)	21.5 (0.0)	13.9 (0.0)	12.8 (0.0)

^a^ Yma_f_, apparent methane yield; Yma_t_, true methane yield; ^b^ Water replacement rate (mL/d); ^c^ Standard deviations are in parentheses.
